# Rényi Entropy in Statistical Mechanics

**DOI:** 10.3390/e24081080

**Published:** 2022-08-05

**Authors:** Jesús Fuentes, Jorge Gonçalves

**Affiliations:** 1 Luxembourg Centre for Systems Biomedicine, University of Luxembourg, Esch-sur-Alzette, L-4367 Luxembourg, Luxembourg; 2 Department of Plant Sciences, Cambridge University, Cambridge CB2 3EA, UK

**Keywords:** statistical mechanics, Rényi entropy, Helmholtz free energy, relative free energy, non-equilibrium thermodynamics, 05.20.-y, 05.30.-d, 05.70.Ln, 03.65.-w

## Abstract

Rényi entropy was originally introduced in the field of information theory as a parametric relaxation of Shannon (in physics, Boltzmann–Gibbs) entropy. This has also fuelled different attempts to generalise statistical mechanics, although mostly skipping the physical arguments behind this entropy and instead tending to introduce it artificially. However, as we will show, modifications to the theory of statistical mechanics are needless to see how Rényi entropy automatically arises as the average rate of change of free energy over an ensemble at different temperatures. Moreover, this notion is extended by considering distributions for isospectral, non-isothermal processes, resulting in relative versions of free energy, in which the Kullback–Leibler divergence or the relative version of Rényi entropy appear within the structure of the corrections to free energy. These generalisations of free energy recover the ordinary thermodynamic potential whenever isothermal processes are considered.

## 1. Introduction

Entropy is one of the most important concepts in physics. It was defined in 1850 by Clausius [1] to refer to the thermodynamic change in the amount of heat $đ\;Q_{rev}$ that is transferred during a reversible process at temperature *T*. Yet, its significance in statistical mechanics belongs to a slightly deeper tier, where it is usually seen as a degree of uncertainty around the state a physical system can reach; or as the bridge between microscopic and macroscopic domains, given it estimates the number of states an atom or molecule shall adopt to fulfil a macroscopic configuration. In addition, because entropy is directly coupled to the second law of thermodynamics, its scope is not limited to statistical treatment but is interrelated to any other area of physics.

In statistical mechanics, entropy is quantified by the Boltzmann–Gibbs measure

(1) $$S=-\sum_{i}^{\Omega }p_{i}\log p_{i},$$

where $p=\{p_{1},\ldots ,p_{\Omega }\}$ is a discrete probability distribution defined for Ω microstates. The particular case p=1/Ω leads *S* to attain its maximum, that is S=−logΩ, which corresponds to an equilibrium state. Boltzmann–Gibbs entropy is additive, since for two non-interacting systems *A* and *B* adequately separated from one another with accessible microstates $\Omega_{A}$ and $\Omega_{B}$, respectively, it follows that $S\left(\Omega_{A}\Omega_{B}\right)=S\left(\Omega_{A}\right)+S\left(\Omega_{B}\right)$. *S* is also extensive provided the entropy of the composite system A+B does satisfy $S\left(\Omega_{A+B}\right)=S\left(\Omega_{A}\right)+S\left(\Omega_{B}\right)$. Of note, the definition of entropy (1) is dimensionless, consequently, throughout this discussion, temperature is to have the dimensions of energy. Then, in order to recover the dimensions of temperature in absolute scale, one shall substitute *T* by $k_{B}T$ into the subsequent formulas, where the conversion factor $k_{B}$ is the Boltzmann constant.

A number of generalisations to Boltzmann–Gibbs entropy have been proposed with a view to pushing the formulation of statistical mechanics to novel boundaries. Some of these attempts have been motivated by deforming the structure of Equation (1), without free parameters, in such a way that the thermodynamic limit remains unaffected [2,3,4]. Whereas other efforts have explored the relaxation of Equation (1) via the introduction of free parameters [5,6]. Among those generalisations, we shall focus on the Rényi entropy, originally introduced in the context of coding and information theory [7] as one of the firsts attempts to broaden Shannon entropy [8], it reads:

(2) $$S_{\alpha }=\frac{1}{1-\alpha }\log\left(\sum_{i}^{\Omega }p_{i}^{\alpha }\right),$$

where α∈R is a deformation parameter, which in the limit α→1 recovers the Boltzmann–Gibbs case, i.e., $\lim_{\alpha \to 1}S_{\alpha }=S$. The logarithmic structure permits that Rényi entropy retains the additive property independently of the value the free parameter assumes, although the extensive property will cease to be preserved for any α≠1.

On account of its features, Rényi entropy has received significant focus in information theory, classical and quantum [9,10,11], provided it is a powerful tool for quantifying quantum entanglement and strong correlations in quantum communications [12]. In multi-partite systems, for instance, the special case α=2 was found to be an intrinsic information measure for the Gaussian states of quantum harmonic oscillators, leading automatically to strong sub-additivity inequalities that entail generalised mutual information measures [13], which computation is tractable through path regularisation schemes [14]. Further applications of Rényi entropy in quantum information include generalisations of conditional quantum mutual information and topological entanglement entropy [15]. As well, in dynamical problems, Rényi entropy has been proposed as a resource to describe phase transitions between self-assembled states, both in complex systems [16,17] and fuzzy systems [18], closely related to the phenomenon of quantum fuzzy trajectories [19]. Still, the feasibility of this entropy measure has even been accepted in other areas, such as molecular imaging pursuing medical purposes [20], mathematical physics [21], and biostatistics [22].

Even though the motivations to introduce entropy (2) were originally nourished by arguments devoid of all physical nature, we are to show that it arises in statistical mechanics in the context of non-isothermal processes. For a system slightly out of thermal equilibrium, the gradient of free energy with respect to the temperature difference may be directly related to the Rényi entropy, see also [23]. In addition, we are to show that in the context of isospectral, non-isothermal processes, it is possible to build relative free energies, both in terms of (1) and (2). These relative free energies have as a special case the ordinary Helmholtz thermodynamic potential whenever the temperatures coincide. In particular, the relative free energy introduced by the relative form of entropy (2) exhibits an additional feature: the parameter α will not only modify the structure of (1), it will also measure the degree to which the probability distributions under consideration are similar.

The discussion will be developed as follows. In Section 2, we will survey fundamental concepts from thermodynamics and statistical mechanics on free energy. In Section 3, we will show that the variation of free energy with respect to temperature leads to Equation (2), where α is defined as the ratio between two temperatures over the interval for which the free energy is defined. In Section 4, we will show that the structure of free energy can be generalised to relative versions by considering two isospectral distributions describing the same system, but at different temperatures. These generalisations are not the result of coincidence, but they follow as free energy is considered in systems out of thermal equilibrium [24,25]. Finally, in Section 5, our conclusions and final thoughts are presented.

## 2. Free Energy

It is well known from thermodynamics that a body subject to an external force field may experience work *W* done on it, bringing the body to a change of its energy state or a change in its configuration. The total change in the body’s energy dE is a conserved quantity and will equal the change of work performed đW plus the heat đQ that the body absorbed from (or transferred to) the medium, where the latter applies if the body is not thermally isolated. Although đW and đQ are inexact differentials, the sum dE=đW+đQ is an exact differential and, therefore, *E* is well-defined in any state, cf. Chapter 2 of Ref. [26].

When the body is confined to a change of state through a reversible process the amount of energy added (or absorbed) in the form of heat đQ equals the infinitesimal change in entropy dS times the temperature *T* (Clausius theorem). In this scenario, the change of work done on a body at constant temperature obeys the equation of state đW=dE−đQ=d(E−TS), where the scalar quantity

(3) $$\begin{array}{c} F=E-TS, \end{array}$$

is known as the Helmholtz free energy. This thermodynamic potential quantifies the amount of work that can be obtained from an isothermal, closed process. Yet, this is not the whole story, in the following sections we are to show that examination of non-isothermal processes across Helmholtz free energy (3) discloses either parametric deformations of *S* in terms of $S_{\alpha }$ or measures of statistical divergence.

As well, from Equation (3) we can look at the infinitesimal change of work available between *F* and F+dF, namely dF=−SdT−PdV, and taking us directly to the well-known relations:

(4) $$-S={\left(\frac{\partial F}{\partial T}\right)}_{V}\;\mathrm{and}\;-P={\left(\frac{\partial F}{\partial V}\right)}_{T}\;.$$

Instead, when the body is treated as a very large ensemble in terms of its internal structure, the formulation of statistical mechanics will necessarily take central stage. Unlike thermodynamics, where there is no probabilistic scheme underlying the theory, in statistical mechanics any measurement to be performed will fetch the sole contribution of the most probable state for which entropy attains its maximum. Thus, if we assume that the ensemble has *N* particles occupying a volume *V* and admits Ω states, then the probability with which the ensemble lies in the energy state $E_{i}=E_{i}\left(N,V\right)$ (i=1,…,Ω) at fixed temperature *T* is given by

(5) $$\begin{array}{c} p\left(E_{i}\right)=\frac{1}{Z}\exp\left(\frac{-E_{i}}{T}\right), \end{array}$$

where $Z=\sum_{i}^{\Omega }\exp\left(-E_{i}/T\right)$ is the partition function, provided the number of particles $\sum_{i}^{\Omega }N_{i}=N$ and the total energy $\sum_{i}^{\Omega }N_{i}E_{i}=E$ are conserved. The explicit form of the energy spectrum does not have any effect on the subsequent discussion.

At this point, it becomes convenient that the Gibbs distribution to be expressed in its canonical form $\omega_{i}=\exp\left(F-E_{i}\right)/T$, such that from the normalisation condition we obtain

$$\sum_{i}^{\Omega }\omega_{i}=\exp\left(\frac{F}{T}\right)\sum_{i}^{\Omega }\exp\left(\frac{-E_{i}}{T}\right)=1,$$

where the partition function is identified with $Z^{-1}=\exp\left(F/T\right)$, therefore, by simply taking logarithms we obtain the fundamental relation in statistical mechanics:

(6) $$\begin{array}{c} F=-T\log Z. \end{array}$$

## 3. Rényi Entropy from Helmholtz Free Energy

We are now in a place to show that the simple study of the partial derivative of Equation (6) with respect to *T*, for fixed *N* and *V*, will lead to entropy (2). We shall start by optimising with respect to the energy spectrum the functional of the form:

$$J=\langle E\rangle +\gamma \sum_{i}^{\Omega }p\left(E_{i}\right)=\sum_{i}^{\Omega }p\left(E_{i}\right)E_{i}+\frac{\gamma }{Z}\sum_{i}^{\Omega }\exp\left(\frac{-E_{i}}{T}\right),$$

where 〈E〉 is the expected value of the total energy, which according to the Gibbs’ postulate equals the thermodynamic energy *E*, and γ is a Lagrange multiplier. To find the optimal values of the energy spectrum, we differentiate *J* with respect to $E_{i}$ and equate to zero, noting that each term must vanish independently, this yields

$$E_{i}=-T\log Z-T\log p\left(E_{i}\right),$$

upon the identification γ=T. Computing the expected value on both sides and dividing by *T*,

$$\frac{\langle E\rangle }{T}=-\log Z+S,$$

using the left formula in (4) for entropy and rearranging terms, we find:

(7) $$\begin{array}{c} {\left(\frac{\partial F}{\partial T}\right)}_{N,V}=-\log Z-\frac{\langle E\rangle }{T}, \end{array}$$

This equation is the expression for free energy in the Gibbs distribution [26]. Of note, the contribution of the expected value of energy in Equation (7) will only be meaningful at temperatures that satisfy the differential inequality

$$-\log Z<-T{\left(\frac{\partial \log Z}{\partial T}\right)}_{N,V}\;,$$

otherwise the behaviour of Equation (7) will be greatly dominated by −logZ. In that regime of temperatures it is true that the inequality ${\left(\partial F/\partial T\right)}_{N,V}<-\langle E\rangle /T$ holds; while at very low temperatures Boltzmann statistics become ineligible, requiring Fermi–Dirac or Bose–Einstein statistics for anti-symmetric or symmetric wave functions, respectively.

We shall assume that small changes in temperature do not give way to sudden jumps in *F*. Such a condition will be fulfilled by demanding *F* to be differentiable at any point of *T*, thus if ${\left(\partial F/\partial T\right)}_{N,V}$ exists at *T* then *F* is necessarily continuous at that point. This also implies that *F* will be continuous for any temperature $T^{\prime }$ arbitrarily close to *T*. Accordingly, we can appeal to the approximation

(8) $${\left(\frac{\partial F}{\partial T}\right)}_{N,V}\approx \frac{F\left(T\right)-F\left(T^{\prime }\right)}{T-T^{\prime }},$$

then, introducing the so-called deformation parameter $\alpha =T/T^{\prime }$, and substituting Equation (6) into the right-hand side of the former expression brings the following outcome:

(9) $$\begin{array}{cc} \frac{F\left(T\right)-F\left(T^{\prime }\right)}{T-T^{\prime }} & =-\frac{T}{T-T^{\prime }}\log\left(\sum_{i}^{\Omega }\exp\left(\frac{-E_{i}}{T}\right)\right)+\frac{T^{\prime }}{T-T^{\prime }}\log\left(\sum_{i}^{\Omega }\exp\left(\frac{-E_{i}}{T^{\prime }}\right)\right)\\ \; & =-\frac{\alpha }{\alpha -1}\log\left(\sum_{i}^{\Omega }\exp\left(\frac{-E_{i}}{T}\right)\right)+\frac{1}{\alpha -1}\log\left(\sum_{i}^{\Omega }\exp\left(\frac{-\alpha E_{i}}{T}\right)\right)\\ \; & =-\frac{1}{\alpha -1}\log Z^{\alpha }\left(T\right)+\frac{1}{\alpha -1}\log\left(\sum_{i}^{\Omega }{\left[\exp\left(\frac{-E_{i}}{T}\right)\right]}^{\alpha }\right)\\ \; & =\frac{1}{\alpha -1}\log\left(\sum_{i}^{\Omega }p^{\alpha }\left(E_{i}\right)\right)\\ \; & =-S_{\alpha }, \end{array}$$

which mirrors the left formula in Equation (4) in terms of the Rényi entropy (2). The deformation parameter α enables upper and lower bounds on the Boltzmann–Gibbs entropy $S=S_{\alpha \to 1}$. This can be seen through differentiation of Equation (2) with respect to α,

$$\frac{\partial S_{\alpha }}{\partial \alpha }=\frac{1}{{\left(1-\alpha \right)}^{2}}\sum_{i}^{\Omega }\pi_{i}\log\left(\frac{p_{i}}{\pi_{i}}\right)\leq 0\;,$$

for any α [16,27], where we have introduced escort probabilities $\pi_{i}=p_{i}^{\alpha }/\sum_{j}^{\Omega }p_{j}^{\alpha }$ and where $\sum_{i}^{\Omega }\pi_{i}\log\left(p_{i}/\pi_{i}\right)$ is known as the Kullback–Leibler divergence [28] (see Section 4). As a consequence, $S_{\alpha }$ decreases monotonically with respect to α, in other words $S_{\alpha }<S_{\beta }$ for β<α, which entails $S_{\alpha \geq 1}\leq S\leq S_{\alpha \leq 1}$, or in terms of Equation (7):

$$S_{\alpha \geq 1}\leq \log Z+\frac{1}{T}\langle E\rangle \leq S_{\alpha \leq 1}.$$

Furthermore, although we have imposed that $T^{\prime }$ is arbitrarily close to *T*, the mean value theorem ensures that Rényi entropy will arise as well if ${\left(\partial F/\partial T\right)}_{N,V}$ exists at some temperature τ whenever it satisfies $T^{\prime }<\tau <T$.

The interesting attribute of free energy shown in Equation (9) also came to Baez’s attention [23], emphasising the unnecessary generalisation of the structure of statistical mechanics for obtaining non-Boltzmannian measures of entropy out of equilibrium. Nevertheless, we shall not omit to say that Equation (9) is defined for non-isothermal processes, in contrast to Equation (7) valid for isothermal processes. Even more, $S_{\alpha }$ may experience abrupt changes in its behaviour due to a lack of stability for any α≠1 given that $S_{\alpha }$ depends on two different temperatures simultaneously. In general, this also is reflected in the impossibility of $S_{\alpha }$ to have a definite thermodynamic limit [29,30], although there exist regions where this entropy is stable [31]. Likewise, Equation (9) fuels further questions about the possibility of obtaining other parametric deformations of entropy without introducing structural generalisations in statistical mechanics. We shall consider this in the next section, but before there is another aspect that is worth commenting on.

Assume $|\sum_{i}^{\Omega }p^{\alpha }\left(E_{i}\right){|}^{2}\leq 1$, the additive property furnished in *S* and $S_{\alpha }$ will be automatically broken by taking the Mercator–Newton series to expand the logarithm in Equation (2), that is

$$S_{\alpha }=\frac{1}{1-\alpha }\log\sum_{i}^{\Omega }p^{\alpha }\left(E_{i}\right)=\frac{1-\sum_{i}^{\Omega }p^{\alpha }\left(E_{i}\right)}{\alpha -1}+\frac{1}{2}\frac{{\left(1-\sum_{i}^{\Omega }p^{\alpha }\left(E_{i}\right)\right)}^{2}}{\alpha -1}+\cdots ,$$

the first term in the series retrieves the Boltzmann-Gibbs entropy by letting α→1. It is a well-known, non-extensive entropy originally derived by Havrda–Charvát [32] in the context of classificatory processes, and, subsequently, introduced by Daróczy [33] in generalised information theory and years after by Tsallis [34] as a possible generalisation of Equation (1) in statistical mechanics.

## 4. Relative Free Energy

We have seen that applying the mean value theorem to the free energy via a deformation parameter that goes as the quotient between the equilibrium and the non-equilibrium temperatures gives rise to Rényi entropy, thus proving that $S_{\alpha }$ has also a direct physical origin. Inherently, Equation (9) hauls the unavoidable question of whether other generalisations are possible without inducing structural changes in the formalism of statistical mechanics while addressing the need to account for phenomena beyond equilibrium [24,25]. This question admits an affirmative answer. As we are to show below, the free energy of a system out of equilibrium can be expressed in terms of the Kullback–Leibler divergence [28] defined as

(10) $$S\left(p,q\right)=\sum_{i}^{\Omega }p_{i}\log\left(\frac{q_{i}}{p_{i}}\right).$$

Even more, when a deformation parameter α is introduced to distort the free energy of a system out of equilibrium we will show that the Rényi relative entropy, which has the form [35]

(11) $$S_{\alpha }\left(p,q\right)=\frac{1}{\alpha -1}\log\left[\sum_{i}^{\Omega }q_{i}{\left(\frac{p_{i}}{q_{i}}\right)}^{\alpha }\right],$$

will result in the expression of relative free energy. The statistical divergence (10) is a special case of (11).

**Definition** **1.** *Let p and q be two isospectral, non-isothermal Gibbs distributions with respective partition functions Z(T) and $Z(T^{\prime })$. The relative free energy for this process is defined as:*

(12) $$F\left(T\right)=-T\log Z\left(T^{\prime }\right)+\left(1-\frac{T}{T^{\prime }}\right){\langle E\rangle }_{p}-TS\left(p,q\right),$$

*where ${\langle E\rangle }_{p}$ is the expected energy with respect to the distribution p.*

**Proof.**  Consider two Gibbs distributions that share the same energy spectrum, albeit at different temperatures, namely $p_{i}=p\left(E_{i},T\right)$ and $q_{i}=q\left(E_{i},T^{\prime }\right)$, whose corresponding partition functions are denoted as Z(T) and $Z(T^{\prime })$. The quotient between these two distributions reads

(13) $$\frac{p_{i}}{q_{i}}=\frac{Z(T^{\prime })}{Z(T)}\exp\left(-\frac{E_{i}}{T}+\frac{E_{i}}{T^{\prime }}\right),$$

after taking logarithms we compute the expectation value on both sides with respect to *p*, then by rearranging terms we obtain

$$\log Z\left(T\right)=\log Z\left(T^{\prime }\right)-\left(\frac{1}{T}-\frac{1}{T^{\prime }}\right){\langle E\rangle }_{p}+S\left(p,q\right),$$

where the last term is the divergence in Equation (10). Finally, using Equation (6) we obtain the formula we want in Equation (12), which is the free energy relative to a configuration that deviates from the equilibrium temperature *T* in $T^{\prime }$ units. That concludes the proof.  □

When the two temperatures coincide, Equation (12) will reduce to Equation (6) since the term of the expected value of energy and the relative entropy S(p,q) will vanish. Even up to a good approximation a comparable regime will emerge for those large ensembles at very near temperatures $T∼T^{\prime }$ for which the relative free energy shall behave closely to Equation (6). As well, in the regime $T\ll T^{\prime }$ the relative free energy will converge to 〈E〉−TlogΩ. Other expressions for relative free energy, but for isothermal processes were studied in [36].

By unfolding these features of statistical mechanics, we shall now probe instances beyond Equation (12). As we are to show, with the following definition, it is possible to obtain a variant of relative free energy but in terms of the Rényi relative entropy defined in Equation (11).

**Definition** **2.** *Let p and q be two isospectral, non-isothermal Gibbs distributions, with partition functions Z(T) and $Z(T^{\prime })$, respectively. Let $\alpha =T/T^{\prime }$ be the deformation parameter, the relative free energy for this process reads explicitly as:*

(14) $$F\left(T\right)=\alpha F\left(T^{\prime }\right)+T^{\prime }\log\left[{\langle \exp\left(-\frac{E}{T^{\prime }}\left(1-\alpha \right)\right)\rangle }_{q}\right]-\left(1-\alpha \right)T^{\prime }S_{\alpha }\left(p,q\right).$$

**Proof.**  Beginning with elevating both sides of Equation (13) to $\alpha =T/T^{\prime }$, gives

$${\left(\frac{p_{i}}{q_{i}}\right)}^{\alpha }={\left(\frac{Z(T^{\prime })}{Z(T)}\right)}^{\alpha }\exp\left[-\alpha E_{i}\left(\frac{1}{T}-\frac{1}{T^{\prime }}\right)\right],$$

multiplying both sides by $q_{i}$ and summing over all accessible states yields

$$\sum_{i}^{\Omega }q_{i}{\left(\frac{p_{i}}{q_{i}}\right)}^{\alpha }=\frac{Z^{\alpha -1}\left(T^{\prime }\right)}{Z^{\alpha }\left(T\right)}\sum_{i}^{\Omega }\exp\left(-\frac{E_{i}}{T^{\prime }}\left(1-\alpha \right)\right)\exp\left(-\frac{E_{i}}{T^{\prime }}\right),$$

after taking logarithms and simplifying the expression, we obtain

(15) $$\begin{array}{cc} S_{\alpha }\left(p,q\right)=\frac{\alpha }{\alpha -1}\log Z\left(T^{\prime }\right) & -\frac{\alpha }{\alpha -1}\log Z\left(T\right)\\ \; & +\frac{1}{\alpha -1}\log\left[{\langle \exp\left(-\frac{E}{T^{\prime }}\left(1-\alpha \right)\right)\rangle }_{q}\right], \end{array}$$

where the left-hand side of the equation is the Rényi relative entropy. Finally, by substituting Equation (6) into Equation (15) and reordering terms, we obtain the formula (14). We have the definition.  □

Equation (14) is the free energy at temperature *T* relative to a configuration at temperature $T^{\prime }$ in terms of the Rényi relative entropy (11). This expression can be seen as a parameterised deformation of Equation (12), which cannot be directly recovered from Equation (14) by simply taking the limit α→1 given the structure of $S_{\alpha }$ would be broken. Certainly, in mathematics and statistics, the functional (11) is often used as a parametric measure of the distance between two probability distributions, which generalises the relative entropy in Equation (10). In this context, nevertheless, Equation (11) will not retrieve the Kullback–Leibler distance since p→q does immediately entail that both temperatures shall coincide, i.e., $T\to T^{\prime }$ and, thus, α→1. In other words, in this representation of Equation (11), the construction of statistical divergence is directly intertwined with the deformation parameter.

Still, in the energy regime $E_{i}\ll T^{\prime }∼T$ Equations (12) and (14) shall behave roughly in the same way. This can be ascertained by noting that in such a regime the expected value of the exponential of energy in Equation (14) can be expanded at first order as

$$\log\left[{\langle \exp\left(-\frac{E}{T^{\prime }}\left(1-\frac{T}{T^{\prime }}\right)\right)\rangle }_{q}\right]\approx \frac{1}{T^{\prime }}\left(1-\frac{T}{T^{\prime }}\right){\langle E\rangle }_{p},$$

while the divergences in Equations (12) and (14) shall approximate each other as

$$\log{\langle \frac{p}{q}\rangle }_{q}∼{\langle \log\frac{p}{q}\rangle }_{p}\;,$$

if p∼q or, equivalently, α∼1.

## 5. Conclusions

We intended this discussion to show that Rényi entropy appears in statistical mechanics without pursuing structural modifications to the theory but by following simple physical arguments. Equations (9) and (14) illustrate the inherent involvement entropy (2) has in statistical mechanics as a result of finding the adequate corrections to free energy in the absence of thermal equilibrium. In general, however, Rényi entropy does not fulfil Lesche’s criterion of stability [29,30], something that may question the physical validity of the relative free energy (14) in that it demands to be well defined. Nonetheless, the observability of Equations (9) and (14) shall not be challenged for thermodynamic systems with a finite number of microstates, as was nicely proved by Jizba and Arimitsu [31], provided some of the instabilities of Rényi entropy are removable upon coarse-grained handling.

We regard Equations (12) and (14) the main contributions of the present discussion to the extent they both reflect how free energy can be described for non-isothermal processes. In particular, (14) takes special attention given that the generalised divergence (11) emerges as a consequence of introducing a deformation parameter $\alpha =T/T^{\prime }$ that distorts the relative free energy (12). This parameterised deformation, however, is completely interrelated with the structure of the relative free energy and Equation (12) shall not be seen as a limit case of Equation (14).

## Data Availability

Not applicable.
